# Advances in Artificial Intelligence and Machine Learning for Precision Medicine in Necrotizing Enterocolitis and Neonatal Sepsis: A State-of-the-Art Review

**DOI:** 10.3390/children12040498

**Published:** 2025-04-13

**Authors:** Miriam Duci, Giovanna Verlato, Laura Moschino, Francesca Uccheddu, Francesco Fascetti-Leon

**Affiliations:** 1 Division of Pediatric Surgery, Department of Women’s and Children’s Health, University of Padova, Via Giustiniani 2, 35128 Padova, Italy; ducimiriam@gmail.com; 2 Pediatric Surgery Unit, Division of Women’s and Children’s Health, Padova University Hospital, 35128 Padova, Italy; 3 Neonatal Intensive Care Unit, Padova University Hospital, 35128 Padova, Italy; giovanna.verlato@aopd.veneto.it (G.V.); laura.moschino@aopd.veneto.it (L.M.); 4 Department of Industrial Engineering, Padova University, 35128 Padova, Italy; francesca.uccheddu@unipd.it

**Keywords:** NEC, artificial intelligence, machine learning, precision medicine

## Abstract

Necrotizing enterocolitis remains one of the most severe gastrointestinal diseases in neonates, particularly affecting preterm infants. It is characterized by intestinal inflammation and necrosis, with significant morbidity and mortality despite advancements in neonatal care. Recent advancements in artificial intelligence (AI) and machine learning (ML) have shown potential in improving NEC prediction, early diagnosis, and management. A systematic search was conducted across multiple databases to explore the application of AI and ML in predicting NEC risk, diagnosing the condition at early stages, and optimizing treatment strategies.AI-based models demonstrated enhanced accuracy in NEC risk stratification compared to traditional clinical approaches. Machine learning algorithms identified novel biomarkers associated with disease onset and severity. Additionally, deep learning applied to medical imaging improved NEC diagnosis by detecting abnormalities earlier than conventional methods. The integration of AI and ML in NEC research provides promising insights into patient-specific risk assessment. However, challenges such as data heterogeneity, model interpretability, and the need for large-scale validation studies remain. Future research should focus on translating AI-driven findings into clinical practice, ensuring ethical considerations and regulatory compliance.

## 1. Introduction

Necrotizing enterocolitis (NEC) remains one of the most devastating gastrointestinal diseases in neonates, characterized by the inflammation and necrosis of the intestinal tissue. Despite advances in neonatal care, NEC continues to have significant morbidity and mortality, particularly among preterm infants. The pathogenesis of NEC is multifactorial, involving complex interactions between immature intestinal immunity, microbial dysbiosis, and environmental factors [1]. Accurate prediction, early diagnosis, and effective management of NEC are crucial and require innovative solutions. Recent advancements in artificial intelligence (AI) and machine learning (ML) have revolutionized the field of neonatal care, offering novel approaches to address these challenges [2]. AI and ML techniques, with their ability to analyze high-dimensional datasets and identify subtle patterns, hold immense potential for improving NEC-related outcomes. These methods have been employed to uncover risk factors, identify non-invasive biomarkers, predict disease onset and progression, and enhance clinical decision making. Additionally, ML algorithms have been utilized to differentiate NEC from conditions with overlapping clinical presentations, such as spontaneous intestinal perforation (SIP) and neonatal sepsis [3].

The effective application of AI in the NEC field depends on a multidisciplinary approach that integrates expertise from neonatology, pediatric surgery, bioinformatics, computational biology, and data science. Multidisciplinary collaboration is essential in precision medicine to bridge the gap between technological advancements and clinical needs. By fostering partnerships among clinicians, researchers, and engineers, it becomes possible to design AI systems that are not only robust and clinically relevant but also ethical and tailored to the complex biological and environmental factors underlying NEC.

This narrative review explores the application of AI and ML in NEC research, focusing on three key domains: (1) identifying risk factors and biomarkers, (2) supporting clinical decision making in NEC management, and (3) distinguishing NEC from SIP and sepsis. By synthesizing the findings from recent studies, this review highlights the transformative impact of ML in neonatal precision medicine and underscores the potential for these technologies to address longstanding challenges in NEC care.

## 2. Materials and Methods

A comprehensive literature search was conducted in PubMed to identify relevant articles published from 2004 to 2024. The search strategy combined keywords and medical subject headings (MeSHs) terms related to NEC, AI, ML, biomarkers, clinical decision making, and neonatal conditions. Example search terms included the following:

- “necrotizing enterocolitis” AND (“artificial intelligence” OR “machine learning” OR “deep learning”);
- “biomarkers” AND “NEC” AND “neonates”;
- “clinical decision support systems” AND “NEC”;
- “spontaneous intestinal perforation” OR “sepsis” AND “machine learning”.

Search results were limited to peer-reviewed articles written in English. The reference lists of relevant studies were also screened to identify additional articles. Studies were included if they performed any of the following:

1. Investigated the use of AI or ML techniques in NEC research or management.
2. Focused on identifying the NEC risk factors, biomarkers, or predictive models.
3. Evaluated ML applications in clinical decision making, such as severity stratification or treatment planning.
4. Explored the differentiation of NEC from SIP, sepsis, or other neonatal conditions.

A total of 45 articles were then selected based on relevance to the scope of the review, with a focus on significant advancements in NEC diagnosis, prediction, and management. “Advancement in NEC field” was defined as studies introducing novel AI/ML applications, identifying new biomarkers, or improving clinical decision-making strategies for NEC differentiation and treatment. The selection process was conducted by the principal investigators, and articles were reviewed collaboratively with other authors to ensure the alignment with the study’s objective. From each included study (26 studies), the following information was extracted: study design and sample characteristics, AI/ML methods employed (e.g., random forest, artificial neural networks, convolutional neural networks), key predictive features, biomarkers, or clinical parameters, model performance metrics (e.g., sensitivity, specificity, AUROC scores), clinical implications, and limitations. We considered NEC definition according to Bell’s stage classification. The extracted data were synthesized to identify recurring themes, emerging trends, and gaps in the current literature. The findings are presented in three main sections corresponding to key applications of AI/ML in NEC research: (1) risk factors and biomarker discovery, (2) clinical decision making, and (3) differentiation from SIP and sepsis. Each section synthesizes the current evidence and discusses the potential impact of these technologies on neonatal care.

### Definitions

Supervised learning: method of training a computer to make predictions or decisions based on labeled data. This means the training dataset includes both input data (features) and the corresponding correct outputs (labels). By analyzing these examples, the algorithm learns the underlying relationship between the inputs and outputs.

Algorithms: linear regression, random forest, decision trees, support vector machines, neural networks., XG-Boost.

Unsupervised learning: a training method of machine learning where algorithms analyze and learn from data that have no labeled outcomes or predefined categories. 

Algorithms: K-means clustering, hierarchical clustering, principal component analysis, density-based spatial clustering of applications with noise.

Deep learning: Deep learning is a type of AI that trains computers to learn and make decisions by looking at lots of examples.

## 3. Results

### 3.1. ML Methods for Determining Risk Factors for NEC

- Patients characteristic and clinical data

The search for non-invasive biomarkers and risk factors for NEC has drawn significant attention, particularly with the application of ML methods. The pioneering study by Mueller et al. in 2009 was the first to apply ML to NEC, utilizing artificial neural networks (ANNs) in a supervised model [4]. From a set of 57 potential risk factors, their model identified being small for gestational age and requiring artificial ventilation as key predictors.

For biomarker discovery, Pantalone et al. analyzed complete blood cell count (CBC) data at various intervals before NEC onset using a random forest (RF) model. While their approach effectively distinguished surgical NEC from controls, the model was less sensitive in differentiating surgical from medical NEC cases [5].

Cho et al. explored NEC prediction using six supervised ML models and a dataset of 74 clinical features. Logistic regression (LR) and RF models emerged as the most effective models, both achieving high accuracy and area under the receiver operating characteristic curve (AUROC) scores. The RF model highlighted 10 essential features, including gestational age, birth weight, sex, multiparity, maternal chorioamnionitis, and treatments such as surfactant and patent ductus arteriosus therapy (medical or surgical ligation), as key differentiators for NEC prediction [6]. Building on these advancements, a recent study developed an AI-based multimodal model that integrates a Residual Network (ResNet34), a deep neural network widely used for image recognition, for imaging analysis, and a one-dimensional convolutional neural network (CNN) for processing laboratory data. The model was trained on a dataset comprising 11,016 laboratory test data points and 408 abdominal X-ray images. This model achieved a high accuracy (94%), sensitivity, and specificity, with an AUC of 0.91, outperforming traditional approaches and performing comparably to experienced clinicians (AUC = 0.83). Additionally, interpretability methods such as GradCAM, a deep learning visualization technique for explaining CNNs, confirmed the model’s ability to focus on clinically significant features like intestinal wall edema and portal venous gas. Its early diagnostic capabilities hold particular promise for improving NEC detection in under-resourced settings, though the further multicenter validation and integration of modalities like abdominal ultrasound are needed to enhance its applicability [7]. Additionally, Feng et al. developed a clinical prediction model for NEC in preterm and very-low-birth-weight infants using multivariate logistic regression. Key predictors identified were temperature, an Apgar score of 5 min, formula feeding, and gestational diabetes mellitus, with the model achieving an accuracy of 82.46% in external validation [8]. Recent advancements have also explored the combination of ML techniques with non-invasive monitoring tools such as near-infrared spectroscopy (NIRS) to improve the NEC risk assessment. Verhoeven et al. investigated the use of NIRS-derived abdominal oxygen saturation (ArSO_2_) levels as an early biomarker for NEC. Their study demonstrated that neonates with an ArSO_2_ <50% within the first day of life had a significantly higher incidence of NEC compared to those with an ArSO_2_ ≥50%. Moreover, the intestinal oxygen extraction fraction was found to be elevated in infants who later developed NEC. By integrating ML algorithms with NIRS data, predictive models could be developed to enhance early NEC diagnosis [9]. The ML-driven analysis of continuous NIRS monitoring could help identify subtle patterns of oxygenation instability that may precede NEC onset, potentially allowing for earlier intervention. This synergy between non-invasive monitoring and AI-based predictive modeling represents a promising direction for improving NEC management, particularly in preterm infants.

- Microbiome and Metabolic Profiling Integration

Microbiome data played a pivotal role in advancing NEC prediction strategies. Hooven et al. employed a multilayer neural network (MIL) model with dimensionality reduction, achieving a high AUROC score and enabling NEC prediction over 24 h before onset [10]. However, the model’s complexity posed challenges in feature interpretation. Lin et al. extended this approach by integrating microbiome profiles with 10 clinical features and an unsupervised MIL model, achieving a prediction window averaging 8.3 days before NEC onset [11].

Subsequent RF analyses identified critical bacterial taxa such as Firmicutes, Proteobacteria, and Enterobacteriaceae as key predictors. Building on these microbiome-based approaches, Olm et al. leveraged taxonomic, metabolic, and bacterial replication data to develop an ML model using gradient-boosted classifiers (GBMs) [12]. Their work narrowed down over 2000 initial features to four key categories, including replication index values, specific secondary metabolite gene clusters, and taxa like Klebsiella, which proved effective for NEC classification. Casaburi et al. corroborated these findings with metagenomic data from multiple studies, demonstrating that NEC-associated bacteria, including Klebsiella pneumoniae and Enterobacter cloacae, were central to their RF model’s decision-making process [13]. Beyond microbiomes, metabolic profiling has been explored as a non-invasive diagnostic tool. Rusconi et al. utilized stool metabolomics and a K-nearest neighbors (KNNs) model, a supervised instance-based learning algorithm used for classification and regression tasks, to identify sphingolipid profiles that effectively distinguished NEC cases from controls, significantly enhancing diagnostic accuracy [14].

### 3.2. ML Methods for Clinical Decision Making in NEC Management

ML algorithms have demonstrated significant promise in stratifying NEC severity, incorporating diverse predictors such as vital signs, laboratory markers, and imaging data using random forest models and implementing decision trees. Recent studies have enhanced these applications by leveraging large-scale neonatal datasets. Ji et al. (2014) developed a data-driven algorithm that integrates clinical and laboratory features to automate the staging and risk stratification of NEC [15]. This tool achieved promising accuracy, with automated Bell’s stage assignments aligning closely with manual clinical assessments (100% accuracy for Stage I and 83.3% for Stage III during testing). Moreover, the study proposed a risk stratification model using linear discriminant analysis (LDA), based on parameters such as pneumatosis intestinalis, portal venous gas, and metabolic acidosis at the onset of the disease, allowing stratification into confidence intervals for medical versus surgical NEC. This method demonstrated an AUC of 0.85 in predicting NEC progression, emphasizing its utility in real-time clinical decision making. Other studies hypothesized that integrated analysis of clinical parameters in combination with urine peptide biomarkers would lead to improved prognostic accuracy in the NEC population. Sylvester et al. examined urinary peptide biomarkers and applied unsupervised ML to isolate peptide clusters that differentiated surgical NEC from medical cases [16]. When integrated with clinical data into a LDA model, their approach outperformed models based solely on clinical features to define the severity of NEC. More recently, Song et al. proposed to use an extensive collection of perinatal, clinical, and laboratory information to assess the prediction of severity of NEC [17]. The application of the advanced ridge regression and Q-learning-based bee swarm optimization (RQBSO) metaheuristic algorithm for differentiating between medical and surgical NEC addresses common challenges in feature selection, such as feature interdependence and redundancy, by integrating a ridge regression approach to enhance efficiency. Specifically, the RQBSO algorithm, a hybrid approach offering robust optimization capabilities in AI-driven healthcare, identified key clinical and laboratory parameters as significant indicators of severe NEC requiring surgery. These include anemia-related factors, mean corpuscular hemoglobin, elevated WBC counts, signs of peritoneal irritation, and the early clinical onset of NEC. Their system achieved remarkable accuracy and AUROC values of 91.88% in the prediction of NEC severity.

- Imaging and Radiographic Analysis

Deep learnings (DL) models have advanced the diagnostic precision and efficiency of radiographic imaging for NEC. In this context, Gao et al. developed a multimodal AI system that integrates radiographic images, clinical data, and laboratory findings to predict surgical NEC [18].

The system demonstrated high accuracy by combining visual data with non-visual predictors, achieving rapid diagnostics suitable for real-time clinical applications. By leveraging CNNs to analyze key imaging features like pneumatosis intestinalis and bowel wall thickening, the model outperformed traditional imaging interpretation in speed and precision. Similarly, deep learning models have been explored for their ability to match a human-level performance in radiographic NEC diagnosis. In a recent study, a ResNet-50 deep convolutional neural network was fine-tuned using transfer learning to identify pneumatosis in neonatal abdominal radiographs. The model achieved an AUROC of 0.918 (95% CI, 0.837–0.978) with an accuracy of 87.8%, performing comparably to senior surgical residents. Gradient-weighted class activation mapping (Grad-CAM) heatmaps confirmed that the network focused on clinically relevant image regions. Notably, senior residents exhibited a median AUROC of 0.896, with individual performance ranging from 0.778 to 0.991, showing no statistically significant difference compared to the AI model. These findings highlight the potential of deep learning systems to support clinicians in accurately and efficiently diagnosing NEC.

Further advancements in AI-driven imaging include the work of Yung et al. that introduced a fine-grained visual classification framework to differentiate NEC severity on radiographs [19]. Utilizing a highly specialized CNN architecture, the system could distinguish between medical and surgical NEC with minimal reliance on manually annotated features. This innovation significantly reduced diagnostic variability and showed potential for integration into neonatal intensive care unit workflows. The study reported an impressive AUROC of 0.94 for surgical NEC classification. DL models can also be used to predict NEC progression by analyzing changes over time in bowel wall integrity, portal venous gas, and free air. This capability can aid in supporting clinical decision making by identifying early signs of surgical NEC, potentially guiding proactive planning for surgical intervention when necessary, rather than relying solely on an algorithm’s decision before clinical signs are evident.

### 3.3. ML Methods to Differentaite NEC from Spontaneous Intestinal Perforation (SIP) and Sepsis

While Ji et al. excluded infants with SIP from their analysis, more recent studies incorporated both SIP and intestinal perforation (IP) in their ML models [15,20,21,22]. Irles et al. utilized backpropagated artificial neural network (ANN) models on two datasets: one comprising 23 neonatal and maternal variables collected at birth and another with 35 variables gathered during hospitalization [20]. Key predictive variables for IP identified by the models included neonatal platelet and neutrophil counts, orotracheal intubation, birth weight, sex, arterial blood gas levels, gestational age, use of fortifiers, patent ductus arteriosus (PDA), maternal age, and maternal health conditions. Similarly, Lure et al. investigated ML approaches to differentiate NEC from SIP, focusing on gestational age at birth, postmenstrual age (PMA) before surgery, and radiographic findings [21]. They identified pneumatosis intestinalis as indicative of NEC, while pneumoperitoneum was associated with SIP. Their models, which included ridge logistic regression and random forest, achieved high AUROC scores, particularly when radiographic data were included, underscoring the importance of imaging in distinguishing these conditions. Son et al. employed various ML algorithms to differentiate between NEC, NEC with IP, and SIP [22]. Their ANN and multilayer perceptron (MLP) models performed the best, with high AUROC scores. In their two-layer model, the first layer separated NEC from NEC with IP, and the second layer distinguished NEC with IP from SIP using the outputs of the first layer. When validated on a new patient dataset, the model achieved AUROC scores ranging from 0.67 to 1.0, with the highest performance (1.0) for predicting NEC with IP and 0.9 for SIP.

Recent advancements in ML have significantly enhanced the ability to distinguish NEC from neonatal sepsis, aiding early diagnosis and intervention. A variety of ML approaches, including ensemble models like random forest and XGBoost, have shown promising results. For instance, stacking classifiers combining XGBoost, random forest, and SVM achieved an impressive 97.04% accuracy in predicting neonatal diseases, including NEC and sepsis, particularly benefiting resource-limited healthcare setting [23,24]. Other studies have developed real-time predictive models for NICUs, such as an XGBoost algorithm that provided hourly risk assessments, detecting severe cases of NEC and sepsis with 81% sensitivity and delivering a median advance of 10 h compared to traditional diagnoses [25]. Additionally, ML models integrating maternal and neonatal features reliably predicted NEC and sepsis morbidities in very-low-birth-weight preterm infants, with AUROCs around 0.73, offering strong potential for advancing neonatal precision medicine [26]. These ML models demonstrate robust potential for integrating patient data and environmental factors to improve neonatal outcomes, showcasing the transformative impact of AI in neonatal care (Table 1).

## 4. Discussion

The incorporation of AI and ML into neonatal care represents a transformative shift in predictive diagnostics, biomarker discovery, and clinical decision making. NEC, a severe gastrointestinal disorder primarily affecting premature infants, remains a challenge due to its multifactorial etiology and the difficulty in early and accurate diagnosis. Recent literature has highlighted that NEC encompasses various forms, not only the one most commonly associated with prematurity. This distinction is crucial, as it may significantly influence the predictive value and accuracy of algorithms used for diagnosing and predicting NEC. The algorithms reviewed in this study may have limitations when applied to non-prematurity-related forms of NEC, and this should be acknowledged as a limitation of our review [27,28].

Before ML’s integration into NEC research, traditional statistical methods like logistic regression and manual analysis of risk factors were the norm. These approaches were often limited by their reliance on small datasets and predefined hypotheses, which often failed to capture the intricate interplay of multifactorial risk factors. Advanced ML algorithms facilitate the integration of heterogeneous data sources, enabling dynamic, data-driven models for early detection and personalized treatment. For instance, hybrid ML models combining clinical and omics data demonstrate promise in forecasting NEC onset up to a week in advance, enhancing preventive care [11,12]. Radiographic interpretation, too, was limited by interobserver variability and the inability to integrate dynamic clinical and laboratory data into predictive models. Automated ML-based tools, incorporating diverse predictors—imaging data, laboratory parameters—achieved higher accuracy and efficiency than manual assessments [18,19,20]. These advancements underscore the transition from traditional clinical staging systems to dynamic, data-driven models capable of adjusting to new insights and datasets. Such systems align with the broader goals of precision medicine, ensuring tailored interventions for NEC management. In addition, the overlap of clinical symptoms in NEC, spontaneous intestinal perforation, and sepsis poses diagnostic challenges. Pre-ML approaches often struggled with overlapping clinical presentations, leading to suboptimal predictive performance and limiting accurate pre-onset forecasts. In contrast, ML algorithms effectively integrate radiographic and clinical data, achieving higher diagnostic accuracy and reducing the median detection time for severe NEC and sepsis by approximately 10 h [21,22]. These advancements underscore the critical role of ML in refining neonatal care strategies using different ML models based on dataset structure, feature availability, and clinical objectives. For example, deep learning techniques excel at recognizing imaging patterns but require extensive labeled datasets, whereas traditional ML models like RF or XGBoost balance interpretability with predictive power. Variations in clinical conclusions across studies arise from differing data sources, sample sizes, and model training approaches. Furthermore, the application of these different ML algorithms could be instrumental in preselecting at-risk patients for perforation associated with NEC, acting as early ‘red flags’ for specific individuals. This early identification could lead to a more rigorous follow-up, involving close clinical monitoring, additional diagnostic tests, and more frequent radiological evaluations. While the decision to act on these predictions before the onset of symptoms would need to be carefully considered, the ability to intervene earlier could facilitate timely and potentially life-saving surgical interventions. In cases where only a short time window exists between the prediction of NEC and the onset of severe NEC, such as the 10 h difference, these algorithms could allow for a more proactive approach, reducing the likelihood of severe outcomes. This shift towards predictive, personalized medicine demonstrates the significant societal value of AI in medical research, potentially improving both patient outcomes and healthcare efficiency.

However, the successful integration of these advanced tools into clinical practice requires targeted efforts in training healthcare professionals and developing new interdisciplinary roles. Training programs should help clinicians to interpret ML outputs and apply them in real-world scenarios. Simultaneously, the emergence of AI in neonatal care calls for the creation of hybrid professional roles, such as clinical data scientists and AI specialists, to bridge the gap between technical innovation and patient care. By fostering such expertise, the healthcare system can ensure that AI-driven tools are effectively leveraged, enhancing both diagnostic precision and patient outcomes.

The transition from traditional to ML-enhanced diagnostic tools signifies a broader shift toward real-time adaptability in neonatal care. Automated models are poised to replace static staging systems, fostering a dynamic approach that accommodates evolving clinical insights and data (Table 2). While data bias exists in both pre-ML and ML approaches, it is essential to emphasize that strategies such as dataset diversification, algorithmic fairness techniques, and rigorous validation processes can be implemented in ML-based methods to mitigate these biases.

### Challenges and Ethical Considerations

Despite these advancements, integrating ML into neonatal care is not without challenges. A key challenge lies in the variability and heterogeneity of neonatal data, including differences in clinical protocols, patient demographics, and microbiome compositions across institutions. These disparities can undermine the generalizability and robustness of AI models. To address this, federated learning—a decentralized approach that trains models across multiple institutions without transferring sensitive data—allows AI to learn from diverse datasets while maintaining privacy and compliance with data protection regulations. Additionally, generative AI techniques, such as generative adversarial networks (GANs), can generate synthetic datasets that represent diverse patient profiles and rare clinical scenarios, enhancing both model robustness and inclusivity.

From an ethical standpoint, the use of sensitive neonatal data raises critical concerns about privacy and data security, particularly given the vulnerability of this patient population. Issues surrounding informed consent are further complicated in emergency situations or when caregivers have a limited understanding of AI technologies. Moreover, biases within training datasets can result in unequal healthcare outcomes, disproportionately affecting underrepresented populations. It is desirable that, with the use of AI in managing all data, robust data protection mechanisms will be implemented.

In addition to these concerns, the transparency of algorithms, the “black-box” nature of AI systems, and the accountability of these models in clinical decision making must be considered. While AI has the potential to improve neonatal care, it is crucial that these systems are transparent, providing clinicians with clear insights into how decisions are made. Ensuring accountability in the event of misdiagnoses or errors is essential, and AI systems should not operate in isolation. Human agency and oversight remain critical in the clinical environment, with healthcare professionals retaining the final responsibility for decisions. Transparency and accountability should be built into AI systems, not only to ensure safety but also to foster trust among patients, caregivers, and healthcare providers.

Combining federated learning with generative AI offers a promising strategy to mitigate these challenges, fostering the development of AI systems that are more equitable, transparent, and generalizable. However, to ensure that these innovations genuinely enhance neonatal care, they must be accompanied by rigorous validation processes and robust ethical frameworks that prioritize patient safety, trust, and human oversight.

## 5. Conclusions

The application of AI and ML in the field of NEC has significantly advanced in research setting to detects its risk factors, interpret biomarkers, and address clinical management strategies. From early diagnostic tools to decision-support systems, these technologies are reshaping neonatal care. Multimodal AI systems that combine clinical, laboratory, radiographic features, omics analysis are particularly promising, offering real-time solutions for complex clinical scenarios. The increasing range of approaches requires a background that goes beyond the sole expertise of clinical professionals. The creation and implementation of these algorithms necessitate collaboration between engineering professionals with expertise in machine learning and clinical experts who understand the specific questions and outcomes they wish to address. This interdisciplinary collaboration ensures that AI systems are both technically robust and clinically relevant. Future research should prioritize the integration of diverse datasets, rigorous model validation, and the development of robust ethical frameworks to guide the responsible application of AI in NEC care. By addressing these challenges, AI-driven innovations can continue to mitigate the burden of NEC and improve outcomes for vulnerable neonatal populations, paving the way for a new era of precision medicine in neonatology.

## Figures and Tables

**Table 1:** Description of ML methods and main findings.

1. ML Methods for Identifying Risk Factors and Biomarkers			
Citation	ML Methods Used	Dataset Size	Key Findings
Mueller et al., 2009 [4]	Artificial neural networks (ANNs)	Small	Identified being small for gestational age and requiring artificial ventilation as key risk factors.
Pantalone et al., 2021 [5]	Random forest (RF)	Small	Used CBC data to distinguish between surgical and medical NEC, with a lower sensitivity in classification.
Cho et al., 2022 [6]	Logistic regression (LR), RF	Large	Identified 10 key features essential for NEC differentiation, including birth weight and gestational age.
Cui et al., 2024 [7]	ResNet34 + CNN (multimodal AI model)	Large	Integrated imaging and laboratory data for NEC diagnosis, achieving 94% accuracy and AUROC of 0.91, outperforming traditional methods.
Feng et al., 2023 [8]	Support vector machines (SVMs), RF	Small	Identified predictors like temperature, Apgar score, formula feeding, and gestational diabetes mellitus.
Verhoeven et al., 2024 [9]	Machine learning + NIRS data analysis	Medium	Identified abdominal oxygen saturation (ArSO_2_) < 50% as a significant NEC predictor. Combined NIRS data with ML to enhance early NEC detection.
Hooven et al., 2020 [10]	Multilayer neural network (MIL)	Small	Predicted NEC 24 h before onset using stool microbiome data.
Lin et al., 2022 [11]	MIL, unsupervised learning	Medium	Integrated microbiome and clinical data, achieving an 8.3-day prediction window before NEC onset.
Olm et al., 2019 [12]	Gradient boosted classifier (GBM)	Medium	Identified metabolic and taxonomic features, such as Klebsiella, as NEC predictors.
Casaburi et al., 2022 [13]	RF	Medium	Used metagenomic data to identify NEC-associated bacteria.
Rusconi et al. 2018 [14]	K-nearest neighbors (KNNs)	Small	Identified stool sphingolipid biomarkers improving NEC diagnosis.
**2. ML Methods for Clinical Decision Making in NEC Management**			
**Citation**	**ML Methods Used**	**Dataset Size**	**Key Findings**
Ji et al., 2014 [15]	Linear discriminant analysis (LDA)	Small	Automated NEC risk stratification, achieving 100% accuracy for Stage I, 83.3% for Stage III.
Sylvester et al., 2014 [16]	Unsupervised ML, LDA	Small	Identified urinary peptide biomarkers, achieving perfect classification when combined with clinical data.
Song et al., 2022 [17]	Ridge regression, Q-learning-based bee swarm optimization (RQBSO)	Small	Differentiated medical vs. surgical NEC, AUROC 0.91.
Gao et al., 2021 [18]	Convolutional neural networks (CNNs)	Medium	Developed a multimodal AI system integrating radiographic images and clinical data.
Weller et al., 2024 [19]	ResNet-50 deep convolutional neural network (DCNN), transfer learning	Small	Achieved AUROC of 0.918, matching senior residents’ performance in pneumatosis detection on NEC radiographs. Used Grad-CAM to confirm model interpretability.
Yung et al. 2024 [20]	CNNs	Medium	Differentiated medical vs. surgical NEC using radiographs, AUROC 0.94.
**3. ML Methods for Differentiating NEC from SIP and Sepsis**			
**Citation**	**ML Methods Used**	**Dataset Size**	**Key Findings**
Irles et al., 2018 [21]	Back-propagated ANN	Small	Identified key predictors for intestinal perforation, including platelet and neutrophil counts.
Lure et al., 2021 [22]	Ridge logistic regression, RF	Small	Identified radiographic differences distinguishing NEC from SIP.
Son et al., 2022 [23]	ANN, multilayer perceptron (MLP)	Large	Achieved AUROC 1.0 for NEC with IP prediction.
Robi et al., 2023 [24]	Stacking classifiers (XGBoost, RF, SVM)	Medium	Achieved high accuracy in predicting NEC and sepsis in neonatal diseases.
Meeus et al., 2024 [25]	XGBoost	Large	Developed a real-time predictive model for NEC and sepsis, detecting severe cases 10 h before clinical diagnosis.
Shu et al., 2024 [26]	XGBoost, RF, logistic regression, SVM, KNN	Large	Predicted NEC and sepsis morbidities in preterm infants, AUROC 0.73.

Legend: Small (<1000 data); Medium (1000–9999 data); Large (≥10,000 data).

**Table 2:** Comparison of pre-machine learning and machine learning approaches: key assumptions, biases, and decision-making implications.

Aspect	Pre-ML Approaches	ML-Driven Advances
Data integration	Isolated biomarkers or single-dimension data	Integrates microbiome, metabolic, clinical, and imaging data
Predictive accuracy	Low	High (AUROC > 0.9 in many cases)
Timeliness	Retrospective or concurrent with clinical events	Predictive capabilities with early warning systems
Clinical decision making	Subjective interpretation, manual tools	Automated, data-driven tools enabling real-time decision-making
Differential diagnosis	Overlapping symptoms of other conditions	High-performing models clearly differentiate related conditions
Human bias	Prone to errors and inconsistencies due to subjective human data entry	ML models standardize interpretation and decision processes
